# Magnesium Hydride Confers Osmotic Tolerance in Mung Bean Seedlings by Promoting Ascorbate–Glutathione Cycle

**DOI:** 10.3390/plants13192819

**Published:** 2024-10-08

**Authors:** Yihua Zhang, Xing Lu, Wenrong Yao, Xiaoqing Cheng, Qiao Wang, Yu Feng, Wenbiao Shen

**Affiliations:** 1 College of Life Sciences, Shanxi Agricultural University, Taigu 030801, China; s20222055@stu.sxau.edu.cn (X.L.); 20232065@stu.sxau.edu.cn (W.Y.); 20201310710@stu.sxau.edu.cn (X.C.); 20201310404@stu.sxau.edu.cn (Q.W.); 20201310422@stu.sxau.edu.cn (Y.F.); 2 College of Life Sciences, Laboratory Center of Life Sciences, Nanjing Agricultural University, Nanjing 210095, China; wbshenh@njau.edu.cn

**Keywords:** osmotic stress, magnesium hydride, hydrogen gas, antioxidant systems, ascorbate–glutathione cycle, mung bean

## Abstract

Despite substantial evidence suggesting that hydrogen gas (H_2_) can enhance osmotic tolerance in plants, the conventional supply method of hydrogen-rich water (HRW) poses challenges for large-scale agricultural applications. Recently, magnesium hydride (MgH_2_), a hydrogen storage material in industry, has been reported to yield beneficial effects in plants. This study aimed to investigate the effects and underlying mechanisms of MgH_2_ in plants under osmotic stress. Mung bean seedlings were cultured under control conditions or with 20% polyethylene glycol (PEG)-6000, with or without MgH_2_ addition (0.01 g L^−1^). Under our experimental conditions, the MgH_2_ solution maintained a higher H_2_ content and longer retention time than HRW. Importantly, PEG-stimulated endogenous H_2_ production was further triggered by MgH_2_ application. Further results revealed that MgH_2_ significantly alleviated the inhibition of seedling growth and reduced oxidative damage induced by osmotic stress. Pharmacological evidence suggests the MgH_2_-reestablished redox homeostasis was associated with activated antioxidant systems, particularly the ascorbate–glutathione cycle. The above observations were further supported by the enhanced activities and gene transcriptional levels of ascorbate peroxidase, monodehydroascorbate reductase, dehydroascorbate reductase, and glutathione reductase. Overall, this study demonstrates the importance of MgH_2_ in mitigating osmotic stress in mung bean seedlings, providing novel insights into the potential agricultural applications of hydrogen storage materials.

## 1. Introduction

Due to climate change and greenhouse gas emissions, future droughts are likely to be more severe, frequent, and persistent than they have been [1,2,3]. As a severe environmental condition, drought induces osmotic stress and then limits plant growth and grain yield by disrupting respiration, photosynthesis, and stomatal movement [4,5]. Additionally, water deficit disturbs the electron transport chain, which, in turn, leads to over-accumulated reactive oxygen species (ROS), such as hydrogen peroxide (H_2_O_2_), superoxide radicals (O_2_·^−^), hydroxyl radicals, and singlet oxygen [6]. Importantly, this excessive ROS accumulation causes oxidative damage to lipids, proteins, and nucleic acids [7,8].

In response to drought and osmotic stress, especially reducing oxidative damage, plants have multiple antioxidant systems to cope with redox imbalance [9]. These antioxidant systems include both enzymatic and non-enzymatic pathways. The enzymatic defense includes catalase (CAT, EC 1.11.3.6), peroxidase (POD, EC 1.11.1.7), and superoxide dismutase (SOD, EC 1.15.1.1), while low-molecular-weight antioxidants, like reduced glutathione (GSH), ascorbate (AsA), carotenoids, and tocopherols, function as non-enzymatic ROS scavengers [10,11]. The ascorbate–glutathione (AsA-GSH) cycle, consisting of AsA, GSH, ascorbate peroxidase (APX, EC 1.11.1.11), monodehydroascorbate reductase (MDHAR, EC 1.6.5.4), dehydroascorbate reductase (DHAR, EC 1.8.5.1), and glutathione reductase (GR, EC 1.8.1.7), plays a pivotal role in detoxifying ROS and maintaining redox homeostasis [4,6]. Ample evidence shows the importance of the AsA-GSH pathway in drought stress responses [12]. For instance, the AsA-GSH cycle was activated, resulting in elevated levels of AsA and GSH and enhanced activities of APX, MDHAR, DHAR, and GR, in order to mitigate oxidative stress caused by water deficiency in *Amaranthus tricolor* and rapeseed plants [13,14].

Hydrogen gas (H_2_) is recognized as a clean and efficient energy source [15]. Previous viewpoints about H_2_ as a biologically inert gas were overturned by an outstanding study by Ohsawa et al. [16], which demonstrated that H_2_ acts as a candidate therapeutic antioxidant, alleviating oxidative damage in animal cells. Further studies have suggested that H_2_ might be a newfound gasotransmitter in animals, plants, and microorganisms [17,18,19,20], although its biosynthetic pathway remains unclear [21]. In plants, H_2_ is associated with multiple abiotic stress responses, such as drought [22], salinity [23], and heavy metal exposure [24]. With growing concerns about severe drought and food security, there has been increasing interest in studying the effects of H_2_ on plant responses to drought and osmotic stress. Studies have shown that H_2_ enhances drought and osmotic tolerance through mechanisms such as increasing proline production, redox homeostasis, and modulating signaling pathways involving nitric oxide, hydrogen sulfide, and abscisic acid [25,26,27,28]. Currently, the most common method for applying exogenous H_2_ in agriculture is traditional electrolytically produced hydrogen-rich water (HRW). This method has shown beneficial effects, such as increasing strawberry yields [29] and improving disease resistance in rice [30]. However, HRW has notable limitations, including the low concentration and short residence time of H_2_ in water [31], which restrict its direct application as an effective plant growth regulator in agriculture.

Magnesium hydride (MgH_2_) is a solid-state metal hydride with a high hydrogen storage capacity (7.6 wt%), lightweight properties, low cost, and the ability to release H_2_ at room temperature [32,33]. The application of MgH_2_ has mainly focused on industry, including hydrogen fuel cells and thermal storage for solar power stations [33,34,35]. It has also been used as an alternative H_2_ donor to prolong the vase life of cut flowers [36,37] and to alleviate copper toxicity in alfalfa [31]. However, the mechanisms of MgH_2_ in plants against drought or osmotic stress remain unclear. In view of previous studies about H_2_ in plants, MgH_2_-regulated drought or osmotic tolerance might be realized through antioxidant systems.

**Figure 1:**
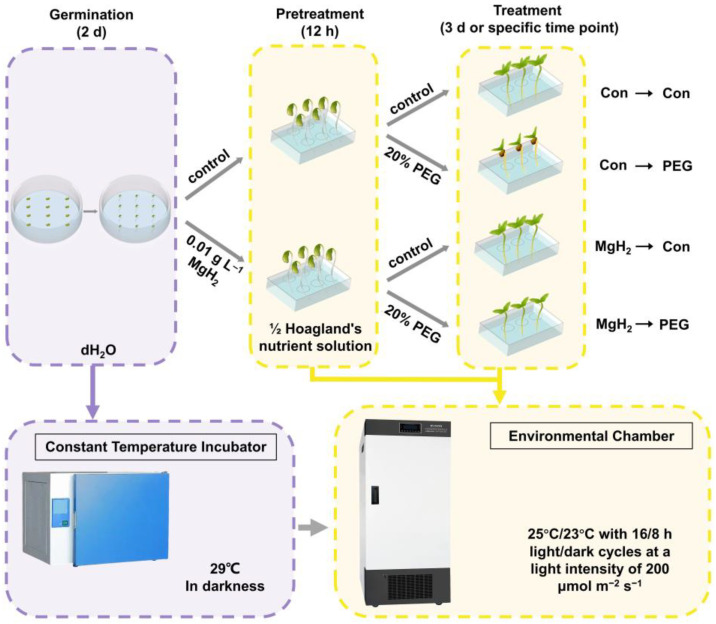
Schematic representation of the experimental setup for this study.

In this study, we set up different experimental groups to investigate the possible function and mechanism of MgH_2_ in mung bean seedlings under osmotic stress (Figure 1). The results show that the MgH_2_-strengthened AsA-GSH cycle played a central role in maintaining redox homeostasis and promoting osmotic resistance. Our study provides insights into the effects of MgH_2_ on osmotic tolerance in mung bean seedlings and introduces a new strategy to support agricultural sustainability and food security.

## 2. Results

### 2.1. Characterization of H_2_ Release in HRW and MgH_2_ Solution

**Figure 2:**
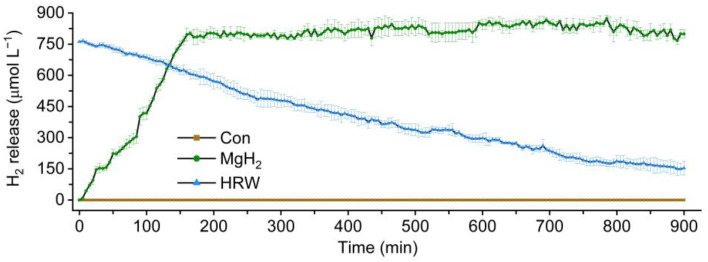
Comparison of H_2_ contents in HRW and hydrolyzed MgH_2_ solution. The means and ± SD values were obtained from three independent experiments with three biological replicates for each.

The operational modes of the HRW preparation and MgH_2_ solution were compared. Notably, the MgH_2_ solution was easier to operate than HRW. Moreover, this study detected the H_2_ level in half-strength Hoagland’s solution (Con), freshly prepared HRW, and MgH_2_ solution. As shown in Figure 2, the content of H_2_ in the MgH_2_ solution was gradually increasing. After reaching its peak value at 3 h, MgH_2_ could maintain its saturation concentration (approximately 800 μmol L^−1^) for at least another 12 h. However, the H_2_ level in HRW decreased to 50% saturation at 3 h. At full saturation, the MgH_2_ solution exhibited a higher H_2_ content and longer retention time than HRW, contributing to more sustained and adequate H_2_ stimulation. Therefore, in subsequent pretreatments, MgH_2_ hydrolyzed for 3 h was used to ensure that the seedlings were stimulated by the high concentration of H_2_.

Due to the hydrolysate of MgH_2_ being H_2_ and magnesium hydroxide (Mg(OH)_2_), the pH changes were also monitored (Figure S1). In our experiment, the pH of half-strength Hoagland’s solution was 6.4. After reacting for 15 h, the addition of 0.01 g L^−1^ of MgH_2_ only slightly increased the pH value to 7.01.

### 2.2. Osmotic Stress-Induced H_2_ Production Was Promoted by MgH_2_

**Figure 3:**
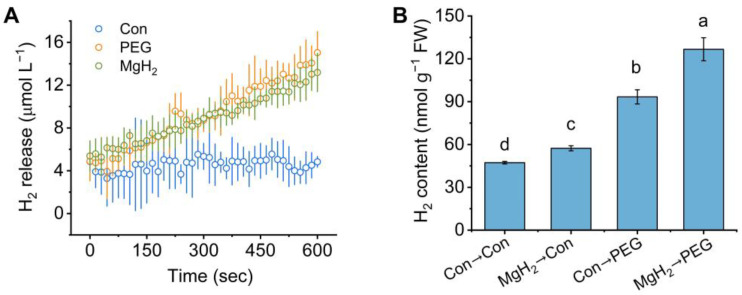
The change in endogenous H_2_ in response to MgH_2_ and PEG treatment. (**A**) Real-time dynamics of H_2_ release from roots of mung bean seedlings in response to PEG (20%) and MgH_2_ (0.01 g L^−1^). (**B**) Endogenous H_2_ accumulation was detected after different treatments by GC. The means and ± SD values were obtained from three independent experiments with three biological replicates for each. The different letters indicate significantly different values (*p* < 0.05) according to Tukey’s multiple range test.

To explore the potential link between osmotic stress, MgH_2_, and endogenous H_2_ metabolism in plants, we investigated the endogenous H_2_ release using a H_2_-specific electrode (Figure 3A). After treatment with PEG or MgH_2_, the endogenous H_2_ release was both rapidly and significantly triggered in the roots of mung bean seedlings in comparison with Con. Subsequently, after pretreatment with or without MgH_2_ and then exposure to either control conditions or polyethylene glycol (PEG)-6000, the endogenous H_2_ content was analyzed using gas chromatography (GC). As shown in Figure 3B, osmotic stress significantly induced H_2_ production in comparison with the control samples. In addition, the H_2_ content was increased by about 21.28% in the samples pretreated with MgH_2_ only. Under osmotic stress, MgH_2_ further elevated the H_2_ content by about 35.70%.

### 2.3. Osmotic Tolerance of Mung Bean Seedlings Achieved by MgH_2_

**Figure 4:**
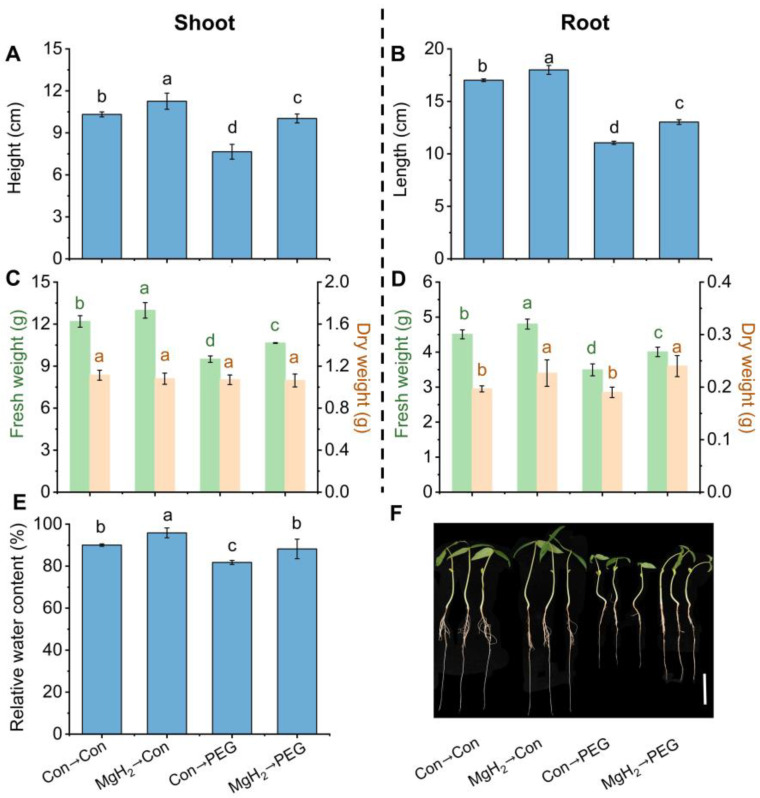
MgH_2_-induced osmotic tolerance. After germinating for 2 days, mung bean seedlings were pretreated with or without 0.01 g L^−1^ of MgH_2_ for 12 h. Subsequently, seedlings were maintained in control conditions (Con) or exposed to osmotic stress (20% PEG) for 3 days. The shoot height (**A**), root length (**B**), FW and DW of 30 shoots (**C**) and 30 roots (**D**), and relative water content (**E**) were detected. (**F**) Representative phenotypes are shown. Scale bar = 5 cm. The means and ± SD values were obtained from three independent experiments with three biological replicates for each. The different letters indicate significantly different values (*p* < 0.05) according to Tukey’s multiple range test.

**Figure 5:**
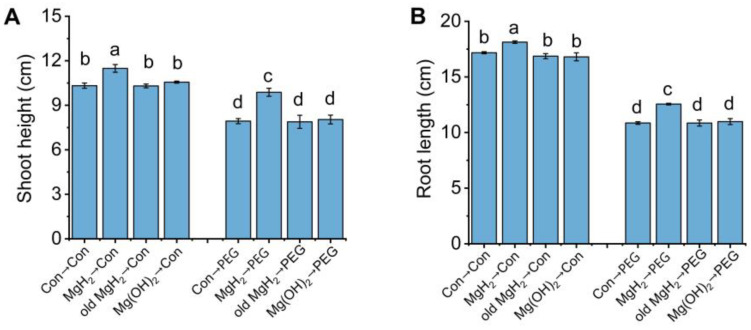
MgH_2_-conferred osmotic tolerance is likely H_2_-dependent. Germinated mung bean seedlings were pretreated with 0.38 mM fresh MgH_2_ (0.01 g L^−1^), old MgH_2_ (losing the ability to release H_2_), or 0.38 mM Mg(OH)_2_ (22.17 g L^−1^) (main by-product of MgH_2_ hydrolysis) for 12 h. The root length (**A**) and shoot height (**B**) were subsequently measured. The means and ± SD values were obtained from three independent experiments with three biological replicates for each. The different letters indicate significantly different values (*p* < 0.05) according to Tukey’s multiple range test.

For the mean trait values, it was observed that MgH_2_ pretreatment significantly enhanced osmotic tolerance, as evaluated by the increases in plant height (Figure 4A), root length (Figure 4B), fresh weight (FW) and dry weight (DW) of shoots (Figure 4C), FW and DW of roots (Figure 4D), and relative water content of the whole seedlings (Figure 4E). The corresponding phenotypes are represented in Figure 4F, which also shows that the growth of mung bean seedlings was obviously inhibited because of osmotic stress; however, this inhibition was significantly alleviated by MgH_2_. Furthermore, the addition of the same concentration of old MgH_2_ (no H_2_ release) or Mg(OH)_2_ (the by-product of MgH_2_ hydrolyzation), two negative controls, showed minor effects on stressed seedlings (Figure 5).

### 2.4. MgH_2_ Conferred Redox Homeostasis in Response to Osmotic Stress

**Figure 6:**
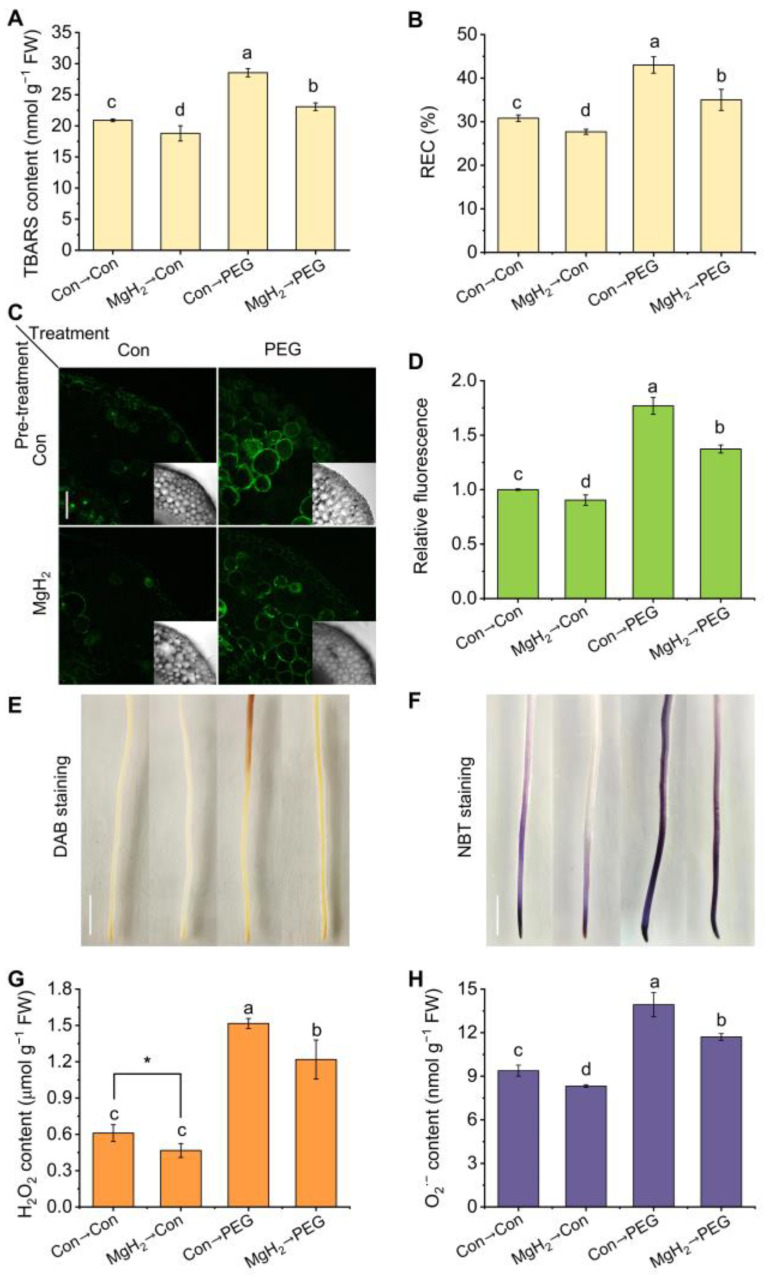
Osmotic stress-induced redox imbalance was reestablished by MgH_2_. After different treatments for 3 days, the contents of TBARS (**A**) and REC (**B**) in roots were determined. The ROS in roots was marked by H_2_DCF-DA and detected by a laser scanning confocal microscope (**C**). Scale bar = 100 μm. The relative fluorescence densities are presented as values relative to Con→Con (**D**). Meanwhile, the H_2_O_2_ and O_2_·^−^ in roots were histochemically stained by DAB (**E**) and NBT (**F**), respectively. Scale bar = 1 cm. The corresponding contents were spectrophotometrically analyzed (**G** and **H**, respectively). The means and ± SD values were obtained from three independent experiments with three biological replicates for each. The different letters and * indicate significantly different values (*p* < 0.05) according to Tukey’s multiple range test or *t*-test.

Osmotic stress-induced lipid oxidative damage is one of the primary causes of plant growth restriction. To evaluate membrane integrity, we assessed the thiobarbituric acid-reactive substance (TBARS) content and relative electrical conductivity (REC). Compared with the control group, the TBARS content and REC in the roots exposed to osmotic stress increased by 36.63% and 39.74%, respectively (Figure 6A,B). In contrast, the above oxidative damage was significantly alleviated by MgH_2_ application, reducing the TBARS content and REC by 19.16% and 18.64%, respectively.

To further investigate the redox status, 2′,7′-dichlorofluorescin diacetate (H_2_DCF-DA, a specific ROS fluorescent probe) was used to monitor endogenous ROS changes, in combination with a laser scanning confocal microscope. As anticipated, osmotic stress significantly triggered fluorescence signals compared with the control group (Figure 6C,D), confirming the occurrence of redox imbalance under osmotic stress. However, stress-triggered fluorescence signals were impaired by MgH_2_, suggesting that MgH_2_ might play a role in redox reestablishment.

Furthermore, the H_2_O_2_ and O_2_·^−^ levels in the roots were visualized using diaminobenzidine (DAB) and nitroblue tetrazolium (NBT) staining, respectively. The results show that H_2_O_2_ (Figure 6E) and O_2_·^−^ (Figure 6F) rapidly accumulated in the roots of stressed seedlings, but this accumulation was prevented after MgH_2_ pretreatment. Similar results were obtained for the H_2_O_2_ (Figure 6G) and O_2_·^−^ contents (Figure 6H).

### 2.5. MgH_2_-Alleviated Oxidative Damage Was Dependent on AsA-GSH Cycle

**Figure 7:**
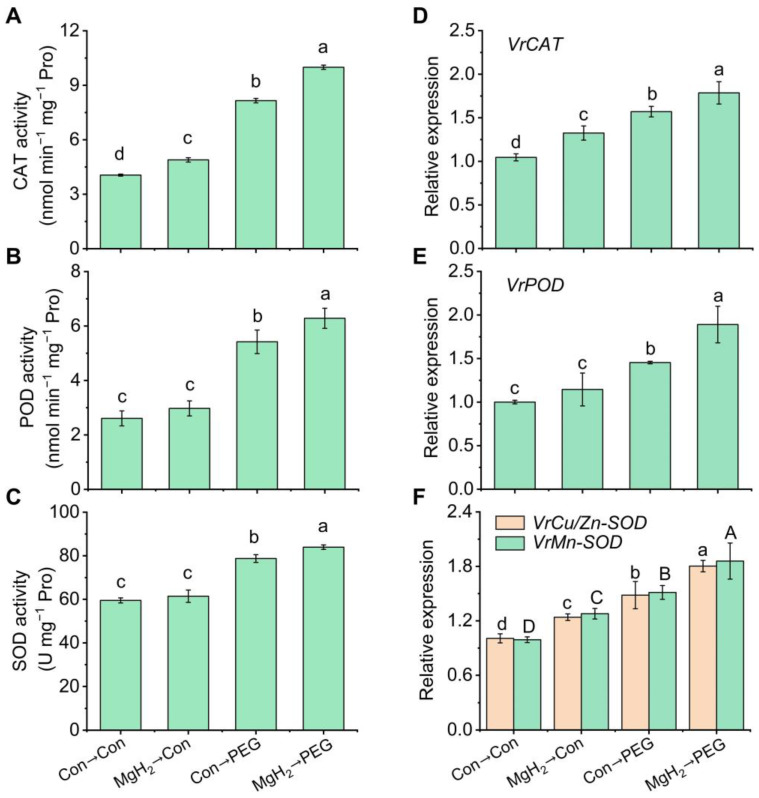
The activities and transcriptional levels of antioxidant enzymes were modulated by MgH_2_ under osmotic stress. After different treatments for 2 days, the activities of CAT (**A**), POD (**B**), and SOD (**C**) in roots were assessed. Following different treatments for 1 day, the corresponding transcriptional levels of *VrCAT* (**D**), *VrPOD* (**E**), and *VrCu/Zn-SOD* and *VrMn-SOD* (**F**) were analyzed. The means and ± SD values were obtained from three independent experiments with three biological replicates for each. The different letters indicate significantly different values (*p* < 0.05) according to Tukey’s multiple range test.

In plants, excessive ROS are mitigated through both enzymatic and non-enzymatic pathways. Our study demonstrated a significant increase in the activities of antioxidant enzymes, such as CAT, POD, and SOD, in response to MgH_2_ application, leading to a reduction in oxidative damage, as illustrated in Figure 7A–C. Furthermore, MgH_2_ also upregulated the expression levels of the corresponding genes under osmotic stress conditions (Figure 7D–F).

**Figure 8:**
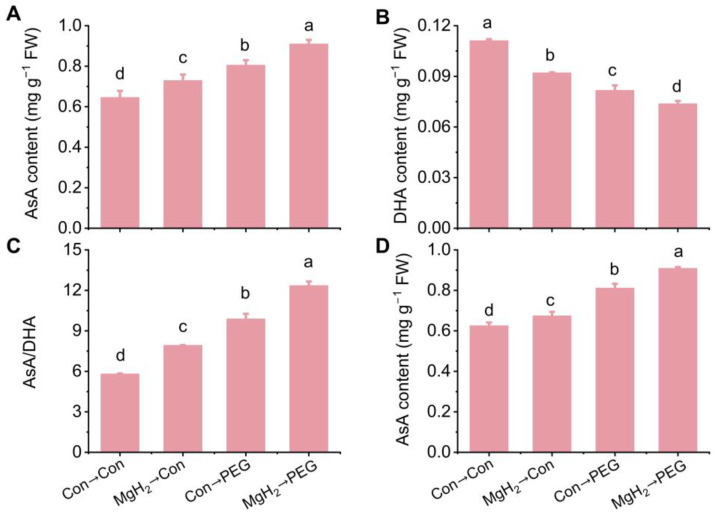
Effects of MgH_2_ on AsA metabolism under osmotic stress. After different treatments for 3 days, the contents of AsA (**A**) and DHA (**B**) in roots were detected. The ratio of AsA to DHA was calculated in (**C**). The AsA content was further validated by HPLC (**D**). The means and ± SD values were obtained from three independent experiments with three biological replicates for each. The different letters indicate significantly different values (*p* < 0.05) according to Tukey’s multiple range test.

To investigate the potential regulation by MgH_2_ of non-enzymatic pathways, the AsA and GSH accumulation were studied. Exposure to osmotic stress elevated the AsA content in the roots of mung bean seedlings (Figure 8A). Pretreatment with MgH_2_ resulted in a significant enhancement in the AsA content under PEG treatment. In contrast, the osmotic stress-decreased dehydroascorbate (DHA, the oxidized form of AsA) content was further reduced by MgH_2_ (Figure 8B). Compared with stress conditions, the ratio of AsA/DHA was further increased after MgH_2_ pretreatment (Figure 8C). Consistently, similar results for the AsA content in the roots were further confirmed by high-performance liquid chromatography (HPLC) (Figure 8D).

**Figure 9:**
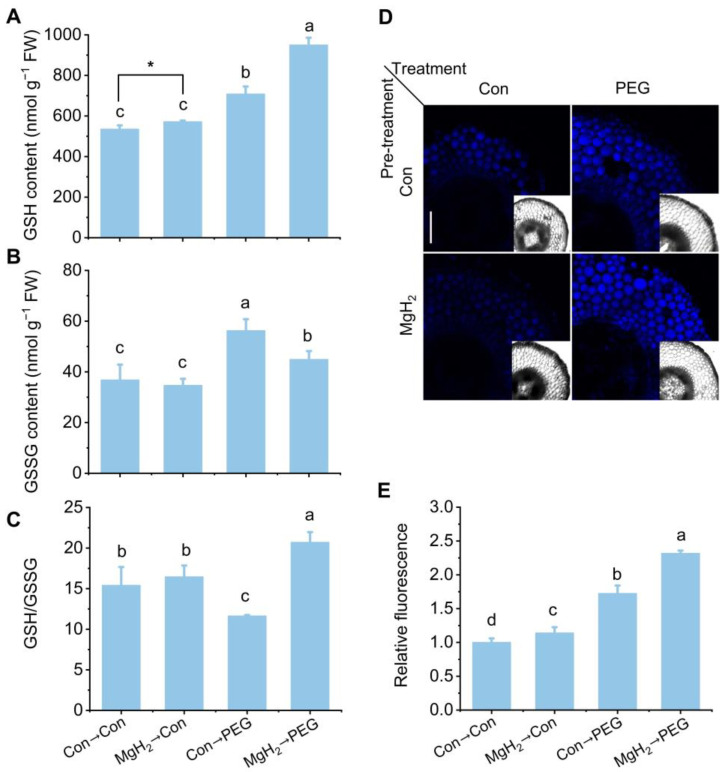
Effects of MgH_2_ on GSH metabolism under osmotic stress. After different treatments for 3 days, the contents of GSH (**A**) and GSSG (**B**) in roots were detected. The ratio of GSH to GSSG was calculated in (**C**). The GSH content was further confirmed by a laser scanning confocal microscope with an MCB fluorescent probe (**D**). Scale bar = 250 μm. Relative fluorescence densities are presented as values relative to Con→Con (**E**). The means and ± SD values were obtained from three independent experiments with three biological replicates for each. The different letters and * indicate significantly different values (*p* < 0.05) according to Tukey’s multiple range test or *t*-test.

Additionally, osmotic stress significantly promoted the GSH (Figure 9A) and oxidized glutathione (GSSG, the oxidized form of GSH) contents (Figure 9B). The MgH_2_ application further increased GSH production but decreased GSSG production. In comparison with the control group, the ratio of GSH/GSSG was suppressed by osmotic stress, but this suppression was reversed by MgH_2_ application (Figure 9C). To further confirm the effect of MgH_2_ on GSH production in the roots, a laser scanning confocal microscope was used to monitor the changes in endogenous GSH, combined with monochlorobimane (MCB, a GSH-sensitive fluorescent probe). As anticipated, the GSH signal in the roots was further intensified by MgH_2_ under osmotic stress (Figure 9D,E).

### 2.6. Regulation of AsA-GSH Cycle-Involved Key Enzymes and Genes by MgH_2_

**Figure 10:**
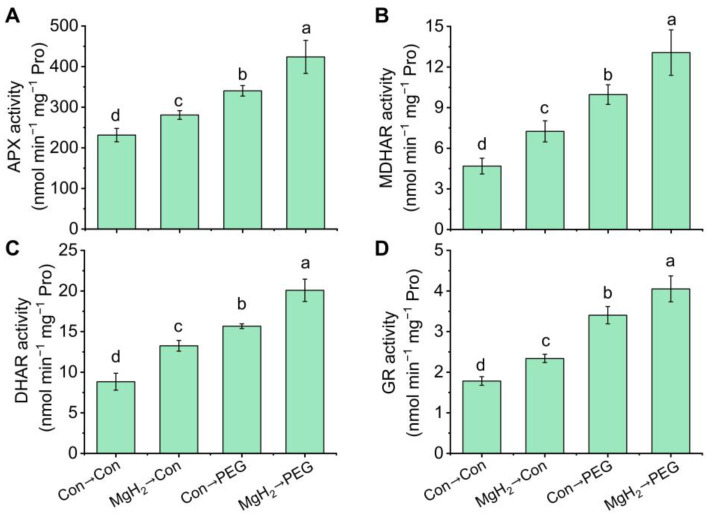
The activities of key enzymes involved in the AsA-GSH cycle modulated by MgH_2_ under osmotic stress. After different treatments for 2 days, the activities of APX (**A**), MDHAR (**B**), DHAR (**C**), and GR (**D**) in roots were analyzed. The means and ± SD values were obtained from three independent experiments with three biological replicates for each. The different letters indicate significantly different values (*p* < 0.05) according to Tukey’s multiple range test.

In order to further verify the above responses, the potential causal link between MgH_2_ and key enzymes involved in the AsA-GSH cycle was investigated. The activities of key enzymes, including APX (Figure 10A), MDHAR (Figure 10B), DHAR (Figure 10C), and GR (Figure 10D), were all increased by osmotic stress. The application of MgH_2_ further enhanced the activities of APX, DHAR, MDHAR, and GR by 24.54%, 28.02%, 31.09%, and 19.12%, respectively.

**Figure 11:**
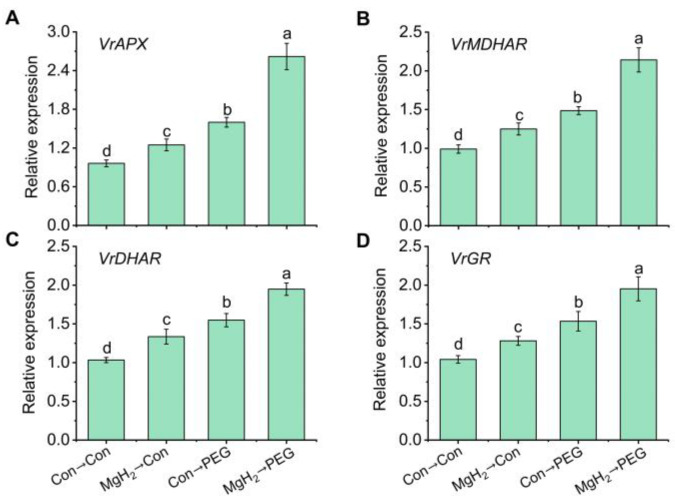
The transcriptional levels of key genes involved in the AsA-GSH cycle regulated by MgH_2_ under osmotic stress. After different treatments for 1 day, the transcriptional levels of *VrAPX* (**A**), *VrMDHAR* (**B**), *VrDHAR* (**C**), and *VrGR* (**D**) in roots were analyzed. The means and ± SD values were obtained from three independent experiments with three biological replicates for each. The different letters indicate significantly different values (*p* < 0.05) according to Tukey’s multiple range test.

Additionally, we assessed the activity of glutathione peroxidases (GPX), an important enzyme involved in GSH biosynthesis. The results show a significant increase of 24.73% in GPX activity following MgH_2_ application under osmotic stress (Figure S2A). Furthermore, the real-time quantitative PCR (qRT-PCR) results show that the transcriptional levels of *VrAPX* (Figure 11A), *VrMDHAR* (Figure 11B), *VrDHAR* (Figure 11C), *VrGR* (Figure 11D), and *VrGPX* (Figure S2B) displayed similar patterns to the changes in their respective enzyme activities.

## 3. Discussion

Growing evidence has shown that H_2_ plays a significant role in modern and sustainable agriculture, particularly in enhancing plant drought and osmotic resistance [38]. Exogenous HRW application has been shown to increase intracellular H_2_ accumulation and reduce stomatal aperture, thereby promoting drought resistance in Arabidopsis [25]. Further studies have indicated that H_2_ regulates drought resistance in alfalfa by modulating the abscisic acid signaling pathway [22,27]. Su et al. [26] also discovered a close correlation between the antioxidant system and exogenous H_2_-mediated osmotic adaptability.

Currently, the main form of H_2_ supply in field agriculture is HRW, which is generated through water electrolysis. However, there are potential risks associated with the overproduction of flammable gas [39]. Additionally, the electrolytic hydrogen generator necessary for HRW preparation is expensive and complex for commercial usage [37]. The lack of reliability and stability also limits the large-scale application of HRW in agriculture [31,40].

In this study, we selected MgH_2_ as a hydrogen-releasing material. Similar to HRW, MgH_2_ application promoted endogenous H_2_ accumulation (Figure 3) and conferred osmotic tolerance to mung bean seedlings (Figure 4), indicating the potential ability of this hydrogen storage material in agricultural production. Recent studies have highlighted the importance of maintaining H_2_ homeostasis for plant growth [31,40]. Compared with electrolytically prepared traditional HRW, the MgH_2_ solution exhibited higher solubility and a longer H_2_ retention time (Figure 2). Most importantly, due to its easy transport and storage, lack of explosion risk, and low cost, MgH_2_ is a more suitable hydrogen-releasing material for application in agriculture.

We also explored the causal link between the by-products of MgH_2_ hydrolysis (primarily Mg(OH)_2_, which leads to increased Mg^2+^ levels and pH) and the growth and stress tolerance of plants. Under our experimental conditions, there were no significant beneficial outcomes in mung bean seedlings with or without the presence of osmotic stress when evaluated with old MgH_2_ or Mg(OH)_2_ [31,36], the two negative controls (Figure 5). These results underline the importance that the above MgH_2_ responses were H_2_-dependent. Although large-scale and long-term field trials are lacking, MgH_2_ appears to be an efficient H_2_-releasing material and may serve as a potential plant growth regulator in agricultural production based on our evaluations. Moreover, we found that MgH_2_ could regulate the physiological activities of other plants, such as cut flowers [36,37] and alfalfa [31], suggesting that its benefits could be extended to other important crops.

In plants, H_2_-mediated redox homeostasis is a crucial pathway for promoting stress resistance [21,38]. As a H_2_ donor, how does MgH_2_ regulate the induction of osmotic tolerance in plants? Our findings clearly indicate that MgH_2_ application mitigated osmotic stress-induced oxidative damage in mung bean seedlings. The evidence for this includes decreased membrane damage (Figure 6A,B) and reestablished redox homeostasis (Figure 6C–H). Combining these findings with previous studies showing MgH_2_’s control of Medicago sativa tolerance against copper stress [31] and the vase life of cut flowers [36,37], we further speculated that MgH_2_’s biological function is based on its antioxidant capability.

To explore the mechanism by which MgH_2_ improves seedling growth inhibition and oxidative damage, we characterized the enzymatic antioxidant pathway. Our data show that antioxidant enzymes, including CAT, POD, and SOD, were upregulated to alleviate oxidative damage under osmotic stress (Figure 7). The above regulation was further reinforced by MgH_2_. Consistently, similar changes in antioxidant enzymes were observed in copper-stressed alfalfa when supplied with MgH_2_ [31]. However, the adaptation of plants facing abiotic stress is only possible if antioxidant enzymes operate in parallel with key antioxidants, providing a great defense capability when redox homeostasis is disrupted [41].

The AsA-GSH cycle is considered a vital part of ROS metabolism in higher plants, with its components primarily located in the cytosol, chloroplasts, peroxisomes, and mitochondria [42]. It is well-documented that AsA and GSH are essential antioxidants in defending against oxidative damage induced by water deficiency in plants [12,43]. In our experiments, PEG treatment resulted in elevated levels of AsA and GSH in the roots of mung bean seedlings compared with the control condition (Figure 8 and Figure 9). These observations were supported by previous findings [13,14]. Importantly, our results reveal that MgH_2_ application further increased the AsA and GSH contents in response to osmotic stress, confirmed through multiple approaches, including spectrophotometry, HPLC, and a laser scanning confocal microscope (Figure 8 and Figure 9). These findings indicate that MgH_2_ serves as a critical regulator in activating the AsA-GSH cycle, subsequently alleviating osmotic stress-induced oxidative damage in mung bean seedlings.

Within the AsA-GSH cycle, AsA functions as a reductant, eliminating H_2_O_2_ via APX, which generates monodehydroascorbate (MDHA) and DHA. The regeneration of AsA from MDHA is catalyzed by MDHAR. The DHA is utilized to generate AsA by DHAR, which accepts electrons from GSH, transferring them into GSSG. Thus, the interplay between the reduction of DHA to AsA and the oxidation of GSH to GSSG is crucial for maintaining the cellular redox state. To keep the GSH pool balance, GR is responsible for regenerating GSH from GSSG. Generally, the cellular redox state (AsA/DHA and GSH/GSSG) and the activity of AsA-GSH cycle-related enzymes (APX, MDHAR, DHAR, and GR) and their corresponding genes’ expression are essential for redox homeostasis and abiotic stress resistance [12,43]. Previous studies have reported that drought and osmotic stress significantly increase the activity of these corresponding enzymes [13,14]. In our study, MgH_2_ pretreatment significantly triggered the activities and transcriptional levels of APX, MDHAR, DHAR, and GR, promoting the efficient recycling of AsA and GSH (Figure 10 and Figure 11), indicating enhanced ROS perception and defense performance against osmotic stress in mung bean seedlings following MgH_2_ application. However, due to the lack of long-term and large-scale field studies, we are still unclear about the potential challenges of MgH_2_ on soil, environment, and plant development. Detailed evaluation results need to be further revealed in the future.

In summary, this study demonstrated that MgH_2_, an efficient H_2_ donor, prevented osmotic stress-induced growth inhibition and oxidative damage in mung bean seedlings. Importantly, MgH_2_-mediated osmotic resistance primarily depends on the elevation in the AsA-GSH cycle and the maintenance of high levels of AsA and GSH.

## 4. Materials and Methods

### 4.1. Plant Materials and Growth Conditions

Healthy and uniform seeds of mung bean (*Vigna radiata* L. Binglv No. 11) were selected and sterilized with 5% (*v/v*) NaClO for 10 min and then washed with distilled water for 1 h. Afterward, the seeds were transferred to clean Petri dishes and germinated in distilled water for 2 d in a constant-temperature incubator set at 29 °C without light.

The two-day-old plants were placed in culture boxes containing 500 mL of half-strength Hoagland’s solution (pH 6.4) and pretreated with or without 0.01 g L^−1^ of MgH_2_ for 12 h. Then, these plants were treated with either half-strength Hoagland’s solution or 20% PEG for an additional 3 days or at specified time points. All seedlings were cultivated in an environmental chamber (25 °C/23 °C with 16/8 h light/dark cycles) at a 200 μmol m^−2^ s^−1^ light intensity in the light stage. The details of the experimental design and main implementation steps of this study are illustrated in Figure 1.

After the treatments, the mung bean seedlings were photographed, and the samples were analyzed immediately or frozen at −80 °C for further analysis.

### 4.2. Detection of H_2_ Release and Content

The H_2_ release in the solution and seedlings was measured using a needle-type Hydrogen Sensor (H2 UniAmp, Unisense, Aarhus, Denmark), following previously reported methods [25,44]. After polarizing for 4 h, the H_2_-specific microelectrode system was employed. The H_2_ release in the Con, saturated HRW (0.78 mM at 25 °C), and MgH_2_ solution (half-strength Hoagland’s solution with 0.01 g L^−1^ of MgH_2_) was detected. The HRW was prepared using a H_2_ generator (SHC-300, Saikesaisi Hydrogen Energy, Jinan, China) in accordance with our previous method [45].

For the endogenous H_2_ detection, a needle was inserted into the root (3 cm from the root tip) to a depth of approximately 200 μm, controlled by a micromanipulator. When the basal line of the H_2_ signal was stable, the treatment solution was added until both the root and electrode tip were immersed, and the corresponding data were recorded. To prevent damage to the electrode tip, the mung bean seedlings were fixed on 2% agarose gel. All manipulations were performed at 25 °C.

To further analyze the endogenous H_2_ content in seedlings, GC (8860 Series, Agilent Technologies, equipped with a thermal conductivity detector) was operated according to a previous report [46] with minor modifications. Briefly, about 0.2 g of the roots was homogenized with 5 mL of distilled water and then placed in a vial, followed by the addition of 200 μL of sulfuric acid (2 M). The air in the vial was displaced by pure nitrogen gas. After being sealed and shaken for 1 min, the vial was heated for 30 min to release the H_2_. Then, the vial was stored at 4 °C before the headspace was analyzed by GC.

### 4.3. Oxidative Damage Assay

The level of oxidative damage was measured by the TBARS content and REC. The amount of TBARS in root tissues was determined based on a previous method [24]. The REC in the roots was analyzed by an electronic conductivity meter (DDSJ-308A, Shanghai Instrument, China), as described previously [47].

### 4.4. Analysis of ROS

The ROS level was determined according to a previous method [48] with minor modifications. After the treatments, approximately 200 μm transversal sections (5 cm from the root tip) were incubated in 25 μM H_2_DCF-DA (a specific ROS fluorescent probe) for 20 min, followed by washing in 20 mM HEPES buffer (pH 7.5) for 30 min. The H_2_DCF-DA signal was observed using a laser scanning confocal microscope (TCS SP8, Leica, Germany), excited at 488 nm and emitted at 500–530 nm. The images were recorded and analyzed by the LAS X v.1.4.7 software (Leica, Wetzlar, Germany). Representative photographs were obtained after the analysis of nine samples for each treatment in three independent experiments.

To visualize the ROS distribution and further assess their content, the H_2_O_2_ and O_2_·^−^ in the roots were histochemically stained and spectrophotometrically analyzed according to previous methods [49].

### 4.5. Determination of Antioxidant Enzyme Activities

For the CAT, POD, and SOD assay, approximately 0.1 g of fresh root tissues was homogenized in 3 mL of 50 mM phosphate buffer (containing 1 mM EDTA and 1% polyvinylpyrrolidone; pH 7.0) at 4 °C. After centrifugation, the supernatant was immediately used for subsequent assays. The protein content in the roots was estimated using a previous method [50]. The activities of CAT, POD, and SOD were measured using an Assay Kit (Suzhou Grace Biotechnology Co., Ltd., Suzhou, China), following the manufacturer’s recommendations.

### 4.6. Detection of AsA and DHA Contents

The AsA and DHA contents were assayed using the procedure described by Kampfenkel et al. [51], with some modifications. Briefly, 2.0 g of the root tissues was homogenized in 5 mL of trichloroacetic acid (TCA; 6%, *w*/*v*) at 4 °C. After centrifugation for 5 min, the supernatant was utilized for analysis. For the AsA determination, 1 mL of the supernatant was transferred to a reaction mixture containing 0.5 mL of potassium phosphate buffer (0.2 M), 0.8 mL of ortho-phosphoric acid (42%, *v/v*), 1.0 mL of TCA (10%, *w*/*v*), 0.8 mL of 2,2′-dipyridyl (4%, *w*/*v*), and 0.4 mL of FeCl_3_ (3%, *w*/*v*). The reaction was incubated at 42 °C for 15 min, and the absorbance was recorded at 525 nm. The total ascorbate (AsA and DHA) content was determined by the reduction of DHA to ASA using dithiothreitol. The DHA content was calculated from the difference between the total ascorbate and AsA contents.

The AsA content was further confirmed by HPLC (U3000, Thermo Fisher Scientific, Waltham, MA, USA) according to a previous method [44]. The root tissues (0.5 g) were homogenized in a TCA solution (0.3 M) and reacted with ascorbate oxidase. After derivatization with 1,2-o-phenylenediamine, the mixture was filtered by a 0.22 μm filter, and then 20 μL of the mixture was injected into the HPLC. The SBC-18 column (4.6 mm × 250 mm; 5 μm particle size; Thermo Fisher Scientific, USA) was maintained at 30 °C. The mobile phase consisted of 0.1 M K_2_HPO_4_-0.08 M KH_2_PO_4_-CH_3_OH (55/25/20, *v/v/v*) at a flow rate of 1.5 mL min^−1^. The AsA content was quantified using fluorescence detection (excitation at 350 nm; emission at 430 nm).

### 4.7. Measurement of GSH and GSSG Levels

The determination of the GSH and GSSG contents followed the method described in a previous publication [52]. Approximately 0.5 g of fresh root tissues was ground in sodium phosphate buffer (50 mM; pH 7.0) at 4 °C. After centrifugation, the supernatant was mixed with 0.1% 2-nitrobenzoic acid. The total glutathione (GSH and GSSG) content was detected at 412 nm. The GSSG content was determined using the same method in the presence of 2-vinylpyridine, and the GSH content was calculated as the difference between the total glutathione and GSSG contents.

The imaging of endogenous GSH in the roots was performed as described previously [53], with minor modifications. Approximately 200 μm transversal sections (5 cm from the root tip) were incubated in 50 μM MCB (a GSH-sensitive fluorescent probe) for 30 min. After washing with HEPES buffer (pH 7.5) three times, the images were observed using a laser scanning confocal microscope (emission at 461 nm and excitation at 380 nm). Representative photographs were obtained after analyzing nine samples for each treatment in three independent experiments.

### 4.8. Detection of Enzyme Activities in AsA-GSH Cycle

The activities of APX, MDHAR, DHAR, and GR were measured following previously established procedures [54], with minor modifications. Approximately 2.0 g of root tissues was homogenized in 10 mL of an extraction buffer (containing 100 mM potassium phosphate buffer, 1 mM AsA, 1 mM EDTA, and 2.5% polyvinylpolypyrrolidone; pH 7.0) at 4 °C. After centrifugation, the supernatant was used immediately to determine the activities of APX, DHAR, MDHAR, and GR.

The APX activity was detected by mixing the supernatant with a reaction solution containing 0.1 mM EDTA, 1 mM AsA, and 1 mM H_2_O_2_. The decrease in AsA was monitored at 290 nm (extinction coefficient: 2.8 mM^−1^ cm^−1^).

The MDHAR activity was estimated by combining the supernatant with a reaction solution that included 0.2 mM NADH, 2.5 mM AsA, and 0.25 units of ascorbate oxidase. The reduction of NADH was monitored at 340 nm (extinction coefficient: 6.2 mM^−1^ cm^−1^).

The DHAR activity was assessed by mixing the supernatant with a reaction solution containing 5 mM GSH and 2 mM DHA. The decrease in DHA was monitored at 265 nm (extinction coefficient: 14.7 mM^−1^ cm^−1^).

The GR activity was determined by mixing the supernatant with a reaction solution consisting of 2.5 mM GSSG, 0.5 mM NADPH, and 1% NaHCO_3_ (*w*/*v*). The oxidation of NADPH was observed at 340 nm (extinction coefficient: 6.2 mM^−1^ cm^−1^).

### 4.9. qRT-PCR Analysis

According to a previous method [28], the total RNA from fresh roots was isolated using the TRIzol reagent (Invitrogen, Waltham, MA, USA), and corresponding reverse transcription was performed by the All-in-One First-Strand Synthesis MasterMix (with dsDNase) (BestEnzymes Biotech Co., Ltd., Lianyungang, China). qRT-PCR was performed with the F488 SYBR qPCR Mix (Universal) (BestEnzymes Biotech Co., Ltd., Lianyungang, China). The primers used for the qRT-PCR are listed in Table S1. *VrActin3* and *VrGAPDH* were selected as the reference genes. The relative gene expression levels were calculated by the formula 2^−ΔΔ*Ct*^ [40].

### 4.10. Statistical Analysis

The data were presented as means ± standard deviations (SD) of three biological replicates. Statistical analyses were performed using the SPSS 18.0 software. The differences between treatments were assessed using a one-way analysis of variance (ANOVA) followed by Tukey’s multiple range test or two-tailed unpaired Student’s *t*-test. Significantly different values were indicated by different letters and an asterisk (*) (*p* < 0.05).

## 5. Conclusions

The use of MgH_2_ presents a more reliable and stable source of H_2_ compared with HRW. In this study, MgH_2_ was found to significantly influence the AsA-GSH cycle, thereby mitigating oxidative damage induced by osmotic stress in mung bean seedlings. These findings deepen our understanding of the role of MgH_2_ in regulating plant physiology and suggest its potential application in future agricultural practices.

## Data Availability

The data are contained within this article and the Supplementary Materials.

## Supplementary Materials

The following supporting information can be downloaded at https://www.mdpi.com/article/10.3390/plants13192819/s1. Table S1: Primers used in qRT-PCR; Figure S1: The kinetic curve of pH value in MgH_2_ solution; Figure S2: The activity of GPX and corresponding gene expression were regulated by MgH_2_ under osmotic stress.
