# Supplementary material for: Magnesium Hydride Confers Osmotic Tolerance in Mung Bean Seedlings by Promoting Ascorbate–Glutathione Cycle
Source: Plants (Basel). 2024 Oct 8;13(19):2819. doi: 10.3390/plants13192819 (PMC11478981; doi:10.3390/plants13192819)
Supplement: Supplementary file 1 [file plants-13-02819-s001.zip › plants-3136736-supplementary.pdf]

Supplementary Materials

Table S1. Primers used in qRT-PCR.

Gene Name	Gene ID	Primer Sequence (5’→3’)
VrCAT	106762083	F: GTATGATCCTGTTCGTCATG
		R: GATTCCTTTGCTAACAGGTG
VrPOD	106755305	F: CGCCGCTGAGAGAAAGAGAA
		R: ATCAACGACACCACAGAGGC
VrCu/Zn-SOD	106764186	F: CGGAAATGGTCCAACAACTG
		R: TGGTCCAGTTGAAAGGCAAC
VrMn-SOD	106752768	F: CTCTGCCGTCGTTAAGCTCC
		R: TGCTGCACCTTCTGCATTAAC
VrAPX	106756347	F: GATGTGTTCGGCAAGGCTATG
		R: CGGACCCTCAAATCCTGAACG
VrMDHAR	106769137	F: ACACGCAACTACAGTGGAGG
		R: CTCCCAAATGGGCAATCCCT
VrDHAR	106769672	F: GGTGCCAGTGGCTCTTTTTG
		R: ACGCTTGCTCTGTTCCATCA
VrGR	106770425	F: GGTGTAGAACAAGGGACGCA
		R: TTGAGCCCTGCTACCAGTTG
VrGPX	106766812	F: TGACAAGGATGGGCAAGTGG
		R: TGACGTTCATCACACGCTCT
VrActin3	106757568	F: ACCTTCAACACTCCTGCTATG
		R: TCGTAAATAGGAACCGTGTGAC
VrGAPDH	106756453	F: TGTGATTTCTGCCCCAAGCA
		R: GAGCAAGGCAATTGGTGGTG

Figure S1.

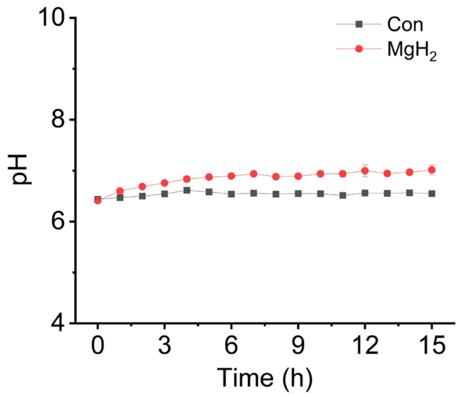

Figure S1. The kinetic curve of pH value in MgH_2 solution. The MgH_2 (0.01 g L^{-1}) were dissolved in half-strength Hoagland's solution (pH 6.4). Afterwards, the pH value was time-dependently recorded by pH meter. The mean and ± SD values were obtained from three independent experiments with three biological replicates for each.

Figure S2.

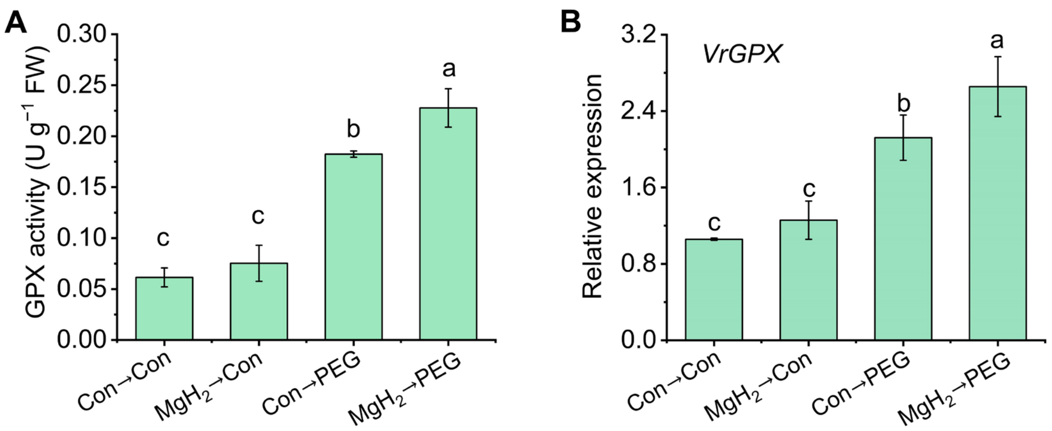

Figure S2. The activity of GPX and corresponding gene expression were regulated by MgH_2 under osmotic stress. After different treatments for 2 days, the activities of GPX (**A**) from roots were detected. After different treatments for 1 day, corresponding transcriptional levels (**B**) was also analyzed. The mean and ± SD values were obtained from three independent experiments with three biological replicates for each. The different letters indicate significantly different values (P < 0.05 according to Tukey's multiple range test).
